# Can We Use Functional Genetics to Predict the Fate of Nitrogen in Estuaries?

**DOI:** 10.3389/fmicb.2020.01261

**Published:** 2020-06-11

**Authors:** Eric J. Raes, Kristen Karsh, Adam J. Kessler, Perran L. M. Cook, Bronwyn H. Holmes, Jodie van de Kamp, Levente Bodrossy, Andrew Bissett

**Affiliations:** ^1^Oceans and Atmosphere, Commonwealth Scientific and Industrial Research Organisation, Hobart, TAS, Australia; ^2^School of Earth, Atmosphere and Environment, Monash University, Melbourne, VIC, Australia; ^3^Water Studies Centre, School of Chemistry, Monash University, Melbourne, VIC, Australia

**Keywords:** denitrification, DNRA, functional genes, estuary, co-occurrence, nitrogen

## Abstract

Increasing nitrogen (N) loads present a threat to estuaries, which are among the most heavily populated and perturbed parts of the world. N removal is largely mediated by the sediment microbial process of denitrification, in direct competition to dissimilatory nitrate reduction to ammonium (DNRA), which recycles nitrate to ammonium. Molecular proxies for N pathways are increasingly measured and analyzed, a major question in microbial ecology, however, is whether these proxies can add predictive power around the fate of N. We analyzed the diversity and community composition of sediment *nirS* and *nrfA* genes in 11 temperate estuaries, covering four types of land use in Australia, and analyzed how these might be used to predict N removal. Our data suggest that sediment microbiomes play a central role in controlling the magnitude of the individual N removal rates in the 11 estuaries. Inclusion, however, of relative gene abundances of *16S*, *nirS*, *nrfA*, including their ratios did not improve physicochemical measurement-based regression models to predict rates of denitrification or DNRA. Co-occurrence network analyses of *nirS* showed a greater modularity and a lower number of keystone OTUs in pristine sites compared to urban estuaries, suggesting a higher degree of niche partitioning in pristine estuaries. The distinctive differences between the urban and pristine network structures suggest that the *nirS* gene could be a likely gene candidate to understand the mechanisms by which these denitrifying communities form and respond to anthropogenic pressures.

## Introduction

The addition of N to aquatic ecosystems has increased markedly over the past 30 years, and is predicted to predominantly increase in estuaries throughout the world in the foreseeable future (Seitzinger et al., 2010; Yu et al., 2019). Eutrophication, attributed to changes in nutrient ratios due to run-off and sedimentation, contributes to numerous negative environmental impacts in estuaries, including loss of habitat and biodiversity, increases in harmful algal blooms, hypoxia and sporadic fish mortalities (Diaz and Rosenberg, 2008; Greaver et al., 2016). In addition, increased anthropogenic nutrient loadings can impact the biogeochemical coupling between the water column and estuarine sediments (Burgin and Hamilton, 2007), which have been shown to further intensify eutrophic conditions (Howarth et al., 2012).

Estuaries and deltas are amongst the most heavily populated and most perturbed parts of the world, with up to 60 percent of the world’s population living along the coast (Small and Cohen, 2004). Estuarine microbial communities are responsible for biogeochemical processes that can remove or recycle N. Unlike other important macro- and micronutrients, e.g., phosphorus and iron (Fe^2+^), the reservoir of bioavailable N is regulated almost solely by biological activity. Microbially mediated processes which regulate the size of the N reservoir include nitrification and denitrification, anaerobic ammonium oxidation (anammox), N_2_ fixation and dissimilatory reduction of NO${}_{3}^{-}$ to NH${}_{4}^{+}$ [DNRA; see Damashek and Francis (2018) and references therein].

The relative importance between N loss and N retention merits investigation due to the adverse effects of excess N in estuarine ecosystems. Denitrification and anammox remove N. DNRA, however, competes with denitrification for available nitrate (NO${}_{3}^{-}$) and retains the reactive N as a more bioavailable form. Understanding the relative importance of N removal (via denitrification) or N retention (via DNRA), including how the balance between these changes under increased N loadings, is key to predict the trajectories of eutrophication in estuarine ecosystems (Giblin et al., 2013). Recently, Kessler et al. (2018) investigated the key controls over the relative rates of denitrification and DNRA in eleven estuaries in Victoria, Australia. Critically, they confirmed several factors which had been identified separately or in incubations, but not together in intact cores. DNRA was found to be enhanced relative to denitrification (1) when nitrate was limiting, consistent with thermodynamic assumptions (Tiedje et al., 1983; Dalsgaard and Bak, 1994); (2) when sediments were highly reducing (e.g., Brunet and Garcia-Gil, 1996); and (3) when large concentrations of dissolved Fe^2+^ were present, consistent with proposed Fe^2+^-DNRA mechanisms (Roberts et al., 2014; Robertson et al., 2016). We expand on the denitrification and DNRA rates measured by Kessler et al. (2018), by integrating molecular data collected in conjunction with the biogeochemical experiments presented therein, obtaining amplicon sequences of the genes catalyzing nitrite reduction during denitrification (*nirS*) and DNRA (*nrfA*). We use these data to test whether we can improve our understanding and prediction capacities for denitrification and DNRA, thereby better understanding drivers of the potential fate of N.

Hypotheses which we tested by incorporating molecular data with the rates from Kessler et al. (2018) were: (H_1_) the same environmental parameters correlated with denitrification and DNRA rates would be correlated with *nirS* and *nrfA* community compositions. (H_2_): based on the functional genes catalyzing nitrite reduction during denitrification and DNRA pathways we could differentiate between estuaries with a low and high N removal capacity. (H_3_): The linear regression models from Kessler et al. (2018) would improve when we integrate bacterial biomass and functional N gene copy data. In summary, here we investigated the potential to use functional gene data to improve/inform the prediction of the fate of N.

## Materials and Methods

### Study Area

Eleven estuaries spanning a range of land uses were sampled along the coast of Victoria, Australia during July and August 2017. Biotic and abiotic samples were collected from the main basin of the estuaries, which were defined as the deepest, central muddy area of the estuary. Water quality and parameters were measured at six depths in the 11 estuaries and a summary of the biochemical and physical metadata can be found in the Supplementary Tables S1–S5 from Kessler et al. (2018) at the following AGU weblink https://agupubs.onlinelibrary.wiley.com/doi/full/10.1029/2018GB005908. DNRA (DNRA15) and denitrification (D15) rates were also measured by Kessler et al. (2018) at six depths. Rates were corrected for site porosity and are expressed in this study in μmol L^–1^ hr^–1^ using total sediment volume. To quantify denitrification and DNRA rates Kessler et al. (2018) used six sediment cores which were sacrificed over six time points; at the start of their rate measurements (referred to as T1; no addition of ^15^N isotope tracer), after 1, 2, 3, 5, and 8 h (last time point is referred to as T6). In this manuscript we present metagenomics data from 6 depths (0–0.5, 0.5–1, 1–2, 2–3, 3–5, and 5–10 cm) and from the time points T1 (environmental representative; *n* = 66 cores) and T6 (enrichment with 50 μmol L^–1^ ^15^NO${}_{3}^{-}$, with a final concentration; *n* = 66 cores) to gain a better insight into the relationships between microbial identity and denitrification and DNRA rates.

The estuaries were classified into four predominant land uses based on percentage of catchment area fertilized (Fert%) and population per km^2^ of catchment area [Pop; see Kessler et al. (2018)]. Overall, three estuaries could be classified as estuaries with high D15:DNRA15 ratios (AIR; WER and PAT) and three could be classified with low D15:DNRA15 ratios (HOP; YAR and MAL). The D15:DNRA15 ratio ranged from estuaries dominated by denitrification (D15:DNRA15 = 8.4) to estuaries dominated by DNRA [D15:DNRA15 = 0.3; as measured by Kessler et al. (2018)].

### DNA Extractions

Sediment cores were collected in polyethylene tubes (6.6 cm internal diameter), and sediment samples were preserved with LifeGuard soil preservation solution (QIAGEN; cat. no. 12868–100) in 50 mL plastic centrifuge tubes and stored at −20°C. The LifeGuard solution was removed after the samples were centrifuged for 5 min at 2,500 *g* and at 4°C. Approximately 2 g of wet sediment was weighed out into the powerbead tubes and DNA and RNA were extracted using the QIAGEN RNeasy PowerSoil Total RNA (QIAGEN; cat. no. 12866-25) and RNeasy PowerSoil DNA Elution (QIAGEN; cat. no. 12867-25) Kits according to manufacturer’s instructions. Nucleic acids were quantified on a QuBIT 2.0 fluorometer.

### Amplicon Sequencing

Amplicons targeting *nirS* (denitrification) and *nrfA* (DNRA) genes were amplified from environmental DNA extracts and sequenced at the Ramaciotti Centre for Genomics (UNSW Sydney). Nextera XT barcode incorporation, purification, library generation and sequencing using the Illumina MiSeq platform (Illumina, Inc., San Diego, CA, United States), with 300 bp paired reads, were performed according to the manufacturer’s directions. PCR primers and cycling conditions for the different amplicons are shown in Table 1.

**TABLE 1:** Library preparation and cycling conditions for the *nirS* and *nrfA* genes.

**PCR cycling conditions**										
**Gene**	**Fw primer**	**Rv primer**	**Initial denaturation**	**Denaturation**	**Annealing**	**Extension**	**Cycles**	**Final extension**	**Hold**	**References**
*nirS*	nirS_Cd3aF	nirS_R3Cd	95°C; 5 min	95°C; 40 s	57°C; 40 s	72°C; 60 s	40	72°C; 10 min	10°C	Michotey et al., 2000; Throbäck et al., 2004
*nrfA*	nrfAF2aw	nrfA_R1	95°C; 5 min	95°C; 40 s	53°C; 45 s	72°C; 60 s	40	72°C; 10 min	10°C	Mohan et al., 2004; Welsh et al., 2014

Zero radius OTU abundance tables were prepared by merging pair-end reads using FLASH (Magoč and Salzberg, 2011), unique sequences were denoised into zero-radius operational taxonomic unit (zOTUs) with the UNOISE algorithm [default settings; Edgar and Flyvbjerg (2015)] using USEARCH 64 bit v8.0.1517 (Edgar, 2010). Per sample abundance profiles were built by mapping all reads per gene against the denoised reads using usearch otutab command (default settings). Amplicon data were analyzed using MEGAN6 (Huson et al., 2007). *nirS* and *nrfA* sequences were aligned against the NCBI-nr database (September 2018) using DIAMOND (Buchfink et al., 2014). Sequence data have been submitted to the National Center for Biotechnology Information under bioproject ID PRJNA61138.

High resolution OTUs (zOTUs, ASVs) are becoming commonly used in microbial molecular surveys, especially those using highly conserved taxonomic marker genes (e.g., *16S* rRNA gene), however, a number of studies have explored different sequence similarity cut-off thresholds ranging from 80 to 100% of protein coding genes to maximize ecological information and minimize noise (Bowen et al., 2013; Caro-Quintero and Ochman, 2015; Lee and Francis, 2017; Tapolczai et al., 2019). In this study we denoised *nirS* and *nrfA* gene sequences into zOTUs and subsequently clustered these into 88% identity threshold OTU’s (using USEARCH -cluster_fast -id 0.88), utilizing both the high and lower resolution data. We acknowledge that the appropriate degree of sequence clustering will vary with the gene investigated, the sequenced region of the gene, and the aims of the research question. By using higher and lower resolution data we have attempted to maximize information and minimize potential noise.

The *nirS* library ranged between 10,162 and 63,322 reads per sample, and the *nrfA* library ranged between 17,677 and 66,449 reads per sample. Pairwise tests, analyses of similarities (ANOSIM), similarity percentage analyses (SIMPER) and canonical correspondence analyses were done on Hellinger transformed, non-rarefied data. Microbiome networks were constructed using rarefied data. Rarefaction curves for the *nirS* and *nrfA* amplicon sequence data are shown in Supplementary Figures S1, S2. As we were interested how the microbial communities were structured in different land uses, we rarified the amplicon data within each land use to its lowest per sample sequence depth, resulting in 14,959 *nirS* reads for the urban sites, 14,491 *nirS* reads for the agricultural sites and 10,162 *nirS* reads for the pristine sites. The rarefied *nrfA* sequence tables for each land resulted in 23,218 reads for the urban sites, 21,496 reads for the agricultural sites and 25,035 reads for the pristine sites (see statistics subsection below for more detail).

### Quantitative PCR

The qPCR reactions were carried out on a 7500 Applied Biosystems real-time PCR instrument. Melt curves were added as a final step in the qPCR reaction to test the stringency of the reactions. PCR products were examined via gel electrophoresis to confirm expected product size specificity. Triplicate, no-template controls did not amplify. Standard deviations between triplicate reactions were <15%. Cycling conditions are shown in Table 2. The gene abundances are expressed as copies per gram wet sediment and data can be found in Supplementary Table S1.

**TABLE 2:** qPCR cycling conditions for the *nirS*, *nrfA*, and *16S* genes.

			**qPCR cycling conditions**									
**Gene**	**Fw primer**	**Rv primer**	**Initial denaturation**	**Denaturation**	**Annealing**	**Extension**	**Fluorescence acquisition**	**Cycles**	**Melt curves**	**Hold**	**Efficiency**	***R*^2^**
*nirS*	nirS_Cd3aF	nirS_R3Cd	95°C; 10 min	95°C; 15 s	57°C; 30 s	72°C; 60 s	80°C; 35 s	40	95°C;15 s 60°C; 1 min 95°C; hold	10°C	80–87%	0.992–0.995
*nrfA*	nrfAF2aw	nrfA_R1	95°C; 10 min	95°C; 15 s	53°C; 45 s	72°C; 60 s	80°C; 35 s	40		10°C	82–95%	0.990–0.997
*B16S*	341f	515R	95°C; 10 min	94°C; 30 s	55°C; 30 s	72°C; 40 s	76°C; 35 s	40		10°C	88–97%	0.991–0.998

### Bacterial *16S* rRNA

Quantitative PCRs of the *16S* rRNA gene were performed using the 341f (CCTACGGGAGGCAGCAG) and 518r (ATTACCGCGGCTGCTGG) primer sets (Muyzer et al., 1993). The 20 μL reaction consisted of 10 μL Power SYBR^TM^ Green PCR Master Mix (Thermo Fisher cat. no. 4368577), 0.1 μL each of 100 μM primer stock 341f and 518r, 0.5 μL BSA and 7.3 μL H_2_O. Standards were created using the *Escherichia coli* non-K-12, wild-type W strain (ATCC #9637) from a Topo cloning kit (cat. number: K204001). Cells were inoculated with lysogeny broth, and DNA was extracted using a DNeasy PowerSoil Kit (Thermo Fisher cat. no. 12888-100). The *E. coli* DNA was then serial diluted from 10 ng μl^–1^ to 10^–5^ ng μl^–1^ to create a standard curve.

### *nirS* qPCR

Quantitative PCRs of the *nirS* gene were performed using the nirS_Cd3aF (GTSAACGTSAAGGARACSGG) and nirS_R3Cd (GASTTCGGRTGSGTCTTGA) primer set (Michotey et al., 2000; Throbäck et al., 2004). The 20 μL reaction consisted of 10 μL Power SYBR^TM^ Green PCR Master Mix (Thermo Fisher cat. no. 4368577), 0.4 μL each of 100 μM primer stock, 0.5 μL BSA and 6.7 μL H_2_O. Standards were created using a *Pseudomonas stutzeri* type strain (DSM 5190; doi: 10.13145/bacdive12996.20180622.3). The *P. stutzeri* DNA was serial diluted from 10 ng μl^–1^ to 10^–5^ ng μl^–1^ to create a standard curve.

### Dissimilatory Nitrate Reduction to Ammonium (DNRA) *nrfA* qPCR

Quantitative PCRs of the *nrfA* gene were performed using primers nrfA_F2aw (CARTGYCAYGTBGARTA) and nrfA_R1 (TWNGGCATRTGRCARTC) from Mohan et al. (2004) and Welsh et al. (2014). The 20 μL reaction consisted of 10 μL Power SYBR^TM^ Green PCR Master Mix (Thermo Fisher cat. no. 4368577), 0.4 μL each of 100 μM primer stock nrfA_F2aw and nrfA_R1, 0.5 μL BSA and 6.7 μL H_2_O. Standards were created using the *E. coli* non-K-12, wild-type W strain (ATCC #9637) from a Topo cloning kit. The *E. coli* DNA was serial diluted from 10 ng μl^–1^ to 10^–5^ ng μl^–1^ to create a standard curve.

### Statistical Analysis

Pairwise tests using permutational multivariate analyses of variances (PERMANOVAs) were used to test whether there were significant differences between the two time points (T1 and T6) within each estuary. Analysis of similarities (ANOSIM) was used to test whether we could identify statistical differences between the 11 estuaries based on the *nirS* and *nrfA* amplicon data clustered at zOTU level and at the 88% similarity level. Similarity percentage analysis (SIMPER) was used to analyze the dissimilarity (%) between the *nirS* and *nrfA* communities for all possible pairwise estuary combinations. Canonical correspondence analyses (CCA) were used to visualize the β-diversity variation in the *nirS* and *nrfA* amplicon data and to identify multiple explanatory variables between the denitrifying (*nirS*) and DNRA (*nrfA*) communities. Environmental parameters were standardized with the ‘standardize’ function (variables were scaled to zero mean and unit variance) using decostand from the Vegan Package (Oksanen et al., 2007) and significant (*p* < 0.05) environmental parameters were derived using the ‘envfit’ function in Vegan and overlaid as vectors. Statistical tests were conducted using the PRIMER v7 software and the Vegan package version 2.5–6 (Oksanen et al., 2007) in R version 3.6.1 (R Core Team, 2013). Co-occurrence networks were made using the OTUs clustered at 88% and zOTU thresholds in R using the igraph version 1.2.4.2 (Csardi and Nepusz, 2006) and qgraph version 1.6.4 (Epskamp et al., 2012) packages. For the networks the OTU tables for the *nirS* and *nrfA* amplicon data were rarefied to the minimum sample depth in each land use, and only the OTUs which contributed >1% were used. Statistical significances of edges were computed in Cytoscape v. 3.7.1 using the CoNet application, as outlined by Faust and Raes (2016). To account for the false discovery rate the *p*-values for the Pearson correlations were computed via 100 permutations and 100 bootstrap distributions. For the permutations we used shuffle_rows as a resampling parameter, and the ‘Renormalize’ option in the CoNet application to reduce the compositionality bias as suggested by Faust et al. (2012), Faust and Raes (2016)). For the regression models we used the same statistical approach as outlined in Kessler et al. (2018), by following the guidelines suggested by Crawley (2012). Correlations and *p*-values were calculated using the Spearman coefficient *r*_s_ using the Corrplot package in R studio. *P*-values were corrected for multiple comparison using the Holm (1979) method.

## Results

### Physical Parameters, Water Quality and Biomass

**FIGURE 1:**
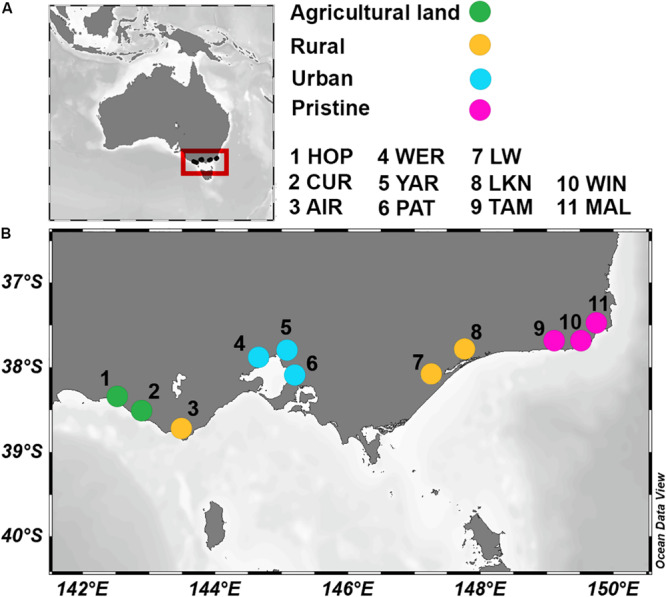
Location of the 11 estuaries in Australia **(A)** and, in greater detail, in the southwestern coast **(B)**, as indicated by the red box in **A**. Colors indicate land usage. Hopkins River (HOP; Fert% 88.7 and Pop 5.5); Curdies River (CUR; Fert% 86.6 and Pop 5.5); Aire River (AIR; Fert% 14 and Pop 1); Werribee River (WER; Fert% 56.4 and Pop 58.3); Yarra River (YAR; Fert% 43.6 and Pop 347); Patterson River (PAT; Fert% 57.1 and Pop 1003); Lake Wellington (LW; Fert% 33 and Pop 11.6); Lake King (LKN; Fert% 14.7 and Pop 2.5); Tamboon Inlet (TAM; Fert% 1.9 and Pop 0.4); Wingan River (WIN; Fert% 0.5 and Pop 0.09); Mallacoota River (MAL; Fert% 2.9 and Pop 0.3).

The 11 sampled estuaries span 5 catchments and could broadly be classified into four land uses. Two agricultural estuaries [HOP and CUR; with a percentage of catchment area fertilized (Fert%) of 87% and a population per km^2^ of catchment area (Pop) of 5.5], three rural areas (AIR, LW, and LKN; were Fert% ranged from 14–33% and Pop from 1 to 11.6), three urban sites (WER, YAR, and PAT; with a Fert% between 43–57% and a Pop from 58 up to 1,003), and three pristine sites (TAM, WIN, and MAL; with a Fert% between 0.5–2.9% and a Pop < 0.4; see Figure 1). Water temperature varied up to 5.6°C between sites, and bottom water salinities ranged from nearly fresh (1.5) to saline (36.4). Oxygen concentrations in the overlying sediment waters ranged from oxygenated (99.4% air saturation) to nearly anoxic (5.6% air saturation). At the pristine sites, NO_x_ and reactive PO${}_{4}^{3-}$ concentrations in the overlying sediment waters were generally ≤1 μmol L^–1^, while highest NO_x_ concentrations were observed at the agricultural site CUR (49 μmol L^–1^). Reactive PO${}_{4}^{3-}$ measured up to 8.0 μmol L^–1^ at the urban site WER. NH${}_{4}^{+}$ concentrations in the waters above the sediment were highest (between 127 and 129 μmol L^–1^) at the agricultural sites (HOP and CUR) and at the urban site (YAR) respectively. Porewater NH${}_{4}^{+}$ concentrations increased with sediment depth and acid volatile sulfide (AVS; a proxy for highly reduced conditions) showed a similar trend. Sediment organic carbon profiles showed similar concentrations from the surface down to 10 cm depth. Principal coordinate analysis (PCoA) of 24 environmental parameters, which included integrated physico-chemical water column and pore water characteristics, showed that each estuary had a unique abiotic signature (see Supplementary Table S2 for 24 physico-chemical parameters and Supplementary Figure S3 for PCoA plot).

The abundances of total bacteria, derived from *16S* rRNA gene qPCRs as a proxy for bacterial biomass, ranged from 2.91 × 10^8^ to 7.85 × 10^9^ *16S* rRNA gene copies per gram wet sediment across the 11 estuaries (Supplementary Table S1). *nirS* gene abundances ranged from 1.58 × 10^6^ up to 6.78 × 10^8^ copies per gram wet sediment, with lowest copy numbers at the surface in the agricultural estuary HOP and highest copy numbers at the surface in the urban estuary YAR (Supplementary Table S1). *nrfA* gene abundances ranged from 1.55 × 10^4^ up to 9.29 × 10^7^ copies per gram wet sediment, with lowest copy numbers at the surface in the rural area LW and highest copy numbers at the surface in the urban estuary YAR (Supplementary Table S1).

### Functional Community Composition

For comparison, sequences were denoised into zOTUs and clustered at 88% identity thresholds (see section “Materials and Methods”). When clustered at 88% sequence similarity, we reduced the number of *nirS* zOTUs from 17,000 to 2,506 OTUs and the number of *nrfA* zOTUs from 37,707 to 13,119 OTUs. No significant differences between T1 and T6 (Monte Carlo tests > 0.1) were noted when the *nirS* and *nrfA* sequences were clustered at a zOTU level nor at the 88% similarity level. Because we did not see any significant differences between the two time points within an estuary, we used both time points in the further analyses.

For both zOTUs and 88% OTUs analysis of similarities (ANOSIM) showed a high and significant separation of the estuaries based on the *nirS* and *nrfA* community data (see Table 3 for the Global *R*- and *p*-values). All pairwise comparisons between the different estuaries for the two N-cycling genes were significantly different (*p* < 0.05). ANOSIM analyses between the four different land uses showed significant differences for the *nirS* and *nrfA* community data (Table 3). The denitrifying community (*nirS*) showed the strong dissimilarity between the different land uses. Again, all pairwise comparisons between the different land uses were significantly different (*p* < 0.05). The lowest ANOSIM values (yet still significant), both for zOTU sequences and sequences clustered at 88% similarity, were found for the *nirS* and *nrfA* communities between estuaries with high and low D15:DNRA15 (Table 3). We used a similarity percentage analysis (SIMPER) to analyze the dissimilarity (%) between the *nirS* and *nrfA* communities for all possible pairwise estuary combinations. The denitrifying communities (*nirS*) in the 11 estuaries were on average 89% different between each other at a zOTU level, and 78% different at the 88% similarity level. SIMPER results for the *nrfA* sequences revealed that the DNRA communities in the 11 estuaries were on average 93 and 82% different at the zOTU and at 88% similarity level, respectively (Supplementary Table S3).

**TABLE 3:** ANOSIM results for two functional N cycling genes with sequences clustered at the zOTU and 88% percent similarity threshold in 11 estuaries in Victoria, Australia.

	***nirS***			***nrfA***		
	**Global *R-*value**			**Global *R-*value**		
	**zOTU**	**88%**	***p*-value**	**zOTU**	**88%**	***p*-value**
Estuary location	0.978	0.986	0.001	0.934	0.897	0.001
Land use	0.535	0.459	0.001	0.445	0.409	0.001
High and low D15:DNRA15	0.183	0.206	0.001	0.144	0.151	0.001

*Global R-values indicate the level of similarity between all sampled sites, with R = 0 indicating strong similarity and R = 1 indicative of a strong dissimilarity. Significant differences at the p < 0.001 level.*

### Environmental Drivers

**FIGURE 2:**
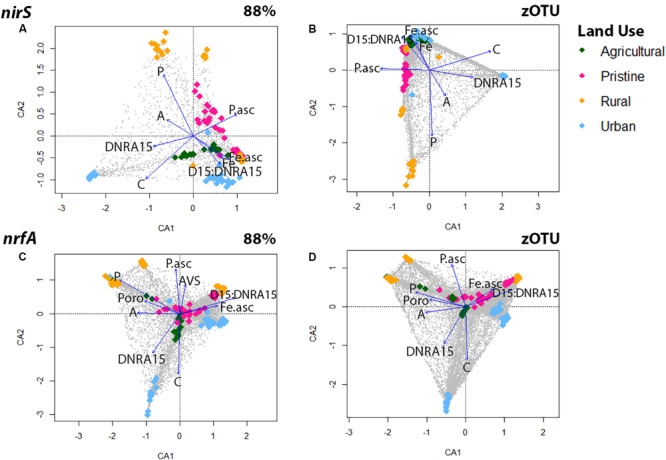
Canonical correspondence analysis (CCA) for the *nirS* gene using OTUs at the 88% similarity **(A)** and zOTU threshold **(B)** and for the *nrfA* gene using OTUs at the 88% similarity **(C)** and zOTU threshold **(D)**. Amplicon data were Hellinger transformed to decrease the contribution of abundant species and environmental parameters were standardized with the ‘standardize’ function using decostand from the Vegan Package (Oksanen et al., 2007). Significant (*p* < 0.05) environmental parameters were derived using the envfit function in Vegan and overlaid as vectors. Environmental parameters from all six depths were: sediment ascorbate-extractible Fe^2+^ concentrations (Fe.asc in μM); Ascorbate extractible (bound) PO${}_{4}^{3-}$ (P.asc in μM); Porosity (Poro); NH${}_{4}^{+}$ in pore water (A in μM); Organic carbon (C in μM); Filterable reactive phosphorus in pore water (P in μM), Acid volatile sulfide (AVS in μM). Colors represent land uses and gray dots are the OTUs.

Correspondence analyses (CCA) were used to explore associations between denitrifying (*nirS*) and DNRA (*nrfA*) community compositions and 24 multiple explanatory variables (Figure 2 and Supplementary Figure S4). Overall, regardless of sequence similarity resolution, the same suit of environmental parameters [dissolved pore water Fe^2+^, NH${}_{4}^{+}$, and PO${}_{4}^{3-}$, organic C, acid volatile sulfide (AVS) and bacterial biomass] showed significant correlations (*p* < 0.05) with the *nirS* and *nrfA* community composition. Organic C and DNRA rates revealed both a positive relationship across the two N-cycling communities, whereas dissolved pore water Fe^2+^ showed a significant and positive effect on estuarine communities with high D15:DNRA15 rates (Figure 2). In detail, the denitrifying communities from five estuaries [the pristine estuary WIN, the agricultural area CUR, the urban sites WER and PAT, and the rural site LKN] were associated with high dissolved pore water Fe^2+^ and high D15:DNRA15 ratios [a proxy for high N removal]. The communities from four estuaries (the pristine sites MAL and TAM, and the rural sites LW and AIR) were associated with high dissolved pore water PO${}_{4}^{3-}$ concentrations and those from the remaining two estuaries [the agricultural site HOP, and the urban site YAR] were associated with high organic C loading and associated higher DNRA rates. CCA analyses for the DNRA communities showed similar results where both the agricultural site HOP, and the urban site YAR were associated with a high organic C loading and higher DNRA rates.

### Network Analyses

The high degree of community dissimilarity, both at the 88% and the zOTU thresholds, between all 11 estuaries suggested that the microbial communities were shaped by their local environment. Microbiome networks based on *nirS* and *nrfA* sequences clustered at the 88% similarity and zOTU thresholds were constructed to provide insights into the microbial community structures for N removal and retention in the urban, agricultural and pristine estuaries. Note, we did not include the rural sites in these analyses as the environmental parameters showed tenfold changes across the three rural sites, the environmental parameters on the other hand for the urban, agricultural and pristine sites were all in the same order of magnitude for each land use.

**FIGURE 3:**
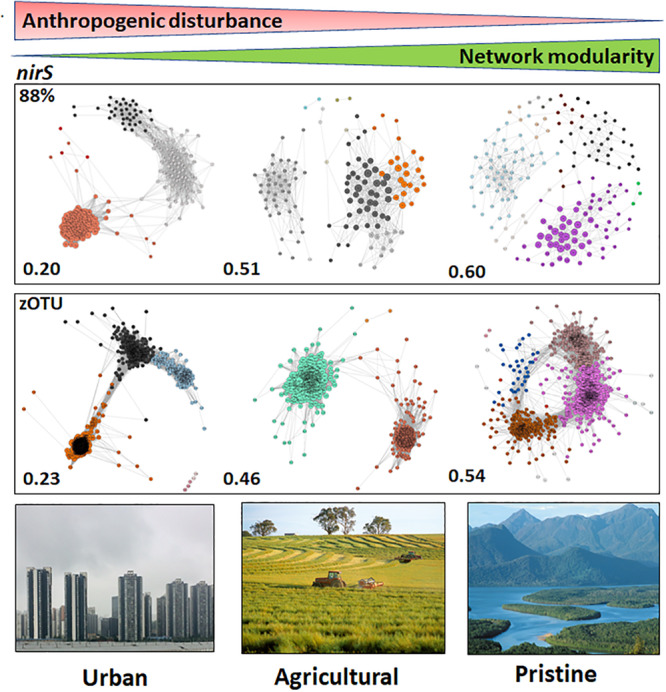
Microbiome networks based on *nirS* sequences clustered at the 88% similarity threshold **(Top)** and the zOTU threshold **(Bottom)** across Urban, Agricultural, and Pristine land uses. Networks were constructed on the OTUs which contributed >1%. For the networks using sequences clustered at the 88% similarity threshold this meant 259 nodes for the urban estuaries, 151 nodes for the agricultural sites and 213 nodes for the pristine sites. For the networks using zOTU sequences this meant 464 nodes for the urban estuaries, 256 nodes for the agricultural sites and 434 nodes for the pristine sites. Only positive co-occurrences with a Pearson correlation *R*^2^ > 0.6 are shown. The Benjamin–Hochberg procedure was used as the multiple testing correction method and only correlations with a corrected *p* < 0.05 are shown. *P*-values of the final network are computed from both 100 permutation and 100 bootstrap distributions, and *p*-values were merged with the Brown method (Brown, 1975). Each circle is a *nirS* OTU (node). Clusters are shown in different colors. Hubs (centrality vectors) are nodes in the network which have a significantly larger number of links compared to the other nodes in the network and are identified by a larger circle diameter. Network modularity for the microbiomes are shown in bold on the left-hand side of the networks. Image credits for the Urban, Agricultural, and Pristine photos: Eric Raes, Gregory Heath, and Willem van Aken, respectively.

The networks based on *nirS* sequences (both at the 88% and zOTU thresholds) showed the highest network modularities in the pristine estuaries, indicating that the networks have dense connections between OTUs within a cluster and sparse connections between clusters. Network modularity decreased with increasing disturbance (increasing human population and anthropogenic pressure), with the urban estuaries showing the lowest network modularities (Figure 3). Keystone OTUs in the *nirS* networks were defined by hub scores > than 0, where a high hub score (max hub score = 1) reflects a significantly larger number of links between OTUs. The urban areas had a higher number of OTUs with a hub score > 0.5 compared to the other two land uses (127 OTUs and 191 zOTUs with a hub score > 0.5). The agricultural and pristine estuaries showed a similar number of OTUs with a hub score >0.5 (32 OTUs and 91 zOTUs with a hub score > 0.5 for the agricultural sites and 20 OTUs and 90 zOTUS with a hub score >0.5 for the pristine estuaries; see Supplementary Figure S5).

**FIGURE 4:**
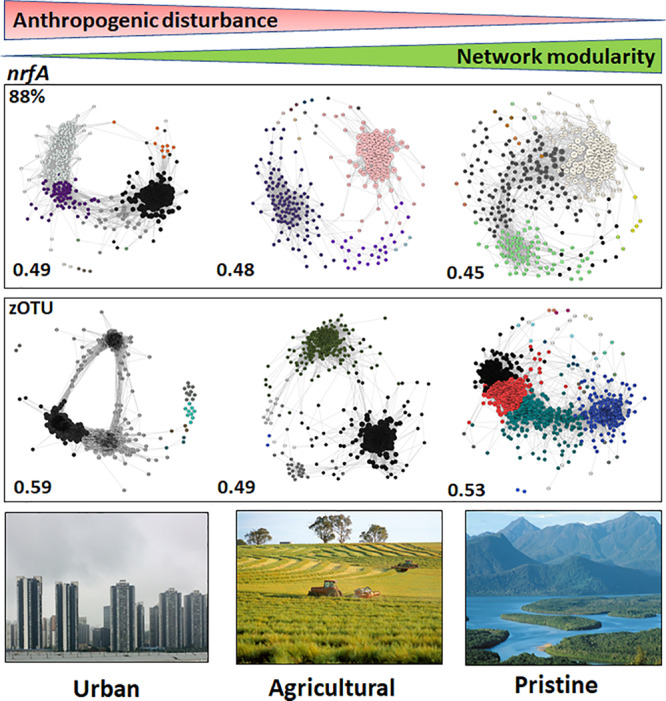
Microbiome networks based on *nrfA* sequences clustered at the 88% similarity threshold **(Top)** and the zOTU threshold **(Bottom)** across Urban, Agricultural, and Pristine land uses. Networks were constructed on the OTUs which contributed >1%. For the networks using sequences clustered at the 88% similarity threshold this meant 441 nodes for the urban estuaries, 309 nodes for the agricultural sites and 433 nodes for the pristine sites. For the networks using zOTU sequences this meant 525 nodes for the urban estuaries, 349 nodes for the agricultural sites and 484 nodes for the pristine sites. Only positive co-occurrences with a Pearson correlation *R*^2^ > 0.6 are shown. The Benjamin–Hochberg procedure was used as the multiple testing correction method and only correlations with a corrected *p* < 0.05 are shown. *P*-values of the final network are computed from both 100 permutation and 100 bootstrap distributions, and *p*-values were merged with the Brown method (Brown, 1975). Each circle is a *nrfA* OTU (node). Clusters are shown in different colors. Hubs (centrality vectors) are nodes in the network which have a significantly larger number of links compared to the other nodes in the network and are identified by a larger circle diameter. Network modularity for the microbiomes are shown in bold on the left-hand side of the networks. Image credits for the Urban, Agricultural, and Pristine photos: Eric Raes, Gregory Heath, and Willem van Aken, respectively.

The modularities of the microbiome networks for the *nrfA* sequence data at 88% similarity did not reveal stark differences between the urban, agricultural and pristine sites (modularities of 0.49; 0.48; and 0.45 for the urban, agricultural and pristine, respectively; Figure 4). At the zOTU threshold the lowest modularity was noted in the agricultural sites (0.49), whereas both the urban (0.59) and agricultural sites (0.53) revealed to have similar modularities. The urban areas had again a higher number of OTUs with a hub score > 0.5 (127 OTUs and 243 zOTUs, respectively). The agricultural areas and the pristine estuaries showed a relative similar number of OTUs with a hub score >0.5 (80 OTUs and 176 zOTUs with a hub score > 0.5 for the agricultural areas and 73 OTUs and 166 zOTUS for the pristine estuaries; see Supplementary Figure S6).

### Modeling DNRA and Denitrification Rates

In order to test whether *16S* rRNA gene and functional N gene abundances could improve model prediction of denitrification rates, DNRA rates and the ratio of D15:DNRA15, we included qPCR data for *16S* rRNA gene (bacterial biomass), and the *nirS* and *nrfA* gene abundance data from the T6 time points to the set of predictor variables used by Kessler et al. (2018) (see Supplementary Table S1 for the predictor variables used in the models). For some models we noted slightly higher *R*^2^ compared to the regression models from Kessler et al. (2018). However, if we use an *a priori* BIC factor [in this case the lowest BIC factor from the model runs by Kessler et al. (2018)], we did not see any improvements compared to the models from Kessler et al. (2018). For this data set, neither the inclusion of *16S*, *nirS*, and *nrfA* qPCR data, nor different ratios of these genes improved the model estimating the ratio of denitrification to DNRA (Supplementary Table S4).

Spearman correlations between the *16S*, the *nirS* and the *nrfA* rRNA gene abundances, the denitrification and DNRA rates and the physico-chemical parameters revealed similar results to the regression and the CCA analyses (Supplementary Figure S7). The *nrfA* rRNA gene abundances showed significant and positive correlations with Fe^2+^, Fe.asc, NH${}_{4}^{+}$ concentrations in the sediment, overlying water NOx concentrations, percentage of catchment area fertilized, and the total copy numbers of bacteria. Neither the *nrfA* rRNA gene abundances nor the *nirS* rRNA gene abundances were significantly correlated with their respective rates. The *16S* rRNA gene abundances were significantly and positively correlated with NH${}_{4}^{+}$ concentrations in the sediment (Supplementary Figure S7).

## Discussion

Understanding the capacity of estuaries to process added N is essential to environmental agencies guiding sustainable development. The fate of N, whether it is removed (via denitrification or anammox) or recycled (via DNRA), becomes, therefore, an important proxy for environmental managers who evaluate the status of estuarine health (Piehler and Smyth, 2011). Direct rate measurements to quantify N removal or N retention are laborious and expensive, making production of data at the resolution required by modelers and decision makers difficult. In this manuscript we tested whether data from functional N genes from 11 temperate estuaries could be used as potential indicators for N removal and retention processes. To provide qualitative and quantitative indicators of microbial nitrogen cycling, we sequenced the *nirS* (shuttling NO${}_{3}^{-}$ to a gaseous form via denitrification) and the *nrfA* genes [shuttling NO${}_{3}^{-}$ to NH${}_{4}^{+}$ via DNRA; Francis et al. (2013)] and, we measured the bacterial biomass and the *nirS* and *nrfA* gene copy numbers. The ratio of denitrification/DNRA can be interpreted as net N removal flux. Previously, Kessler et al. (2018) suggested that N removal via anammox was negligible in these samples, hence we refer to N removal in the further discussion as denitrification.

### Environmental Parameters

Our first hypothesis proposed that the environmental parameters previously correlated with denitrification and DNRA rates would be correlated with their respective community compositions. In order to see whether differences in sequence resolution impacted the correlations of the environmental parameters we utilized both zOTUs and the 88% OTUs for comparison. zOTUs provide higher resolution, which may improve the ability to detect biogeographical patterns, such as strain-specific preferences for environmental parameters (Callahan et al., 2017). On the other hand, clustering at 88% similarity reduces the data complexity and may reveal whether ecological trends hold up at a coarser level, as shown by Bowen et al. (2013) and Lee and Francis (2017) for the *nirS* gene. Regardless of resolution, our data showed that the microbial communities catalyzing N removal and N recycling processes were statistically distinct in each of the 11 estuaries. At a zOTU level there were little differences in the Global *R*-values (the measure for the dissimilarity between estuaries) based on *nirS* and *nrfA* gene data. In contrast, while the *nrfA* data clustered at 88% similarity maintained a relative high alpha diversity compared to the *nirS* data, the *nrfA* community showed overall lower Global *R*-values compared to the *nirS* data.

Importantly, the same physico-chemical factors (C, PO${}_{4}^{3-}$, Fe^2+^, AVS, NH${}_{4}^{+}$, and porosity) that correlated most strongly with N community composition (this study) are those that correlate most strongly with direct rate measurements of denitrification and DNRA and net N removal [denitrification:DNRA; Kessler et al. (2018)]. These findings were similar for both clustering thresholds. This suggests a connection between physico-chemical factors, N community composition, and biogeochemical rates: a requirement for the use of genetic information as a proxy for rates or relative rates. The emergence of shared environmental drivers between N community composition and the relevant biogeochemical rates is compelling in a sample set such as this one; single samples collected from disparate estuaries with widely varying inputs of nutrients and organic C.

The findings that the *nirS* denitrifying communities could be roughly divided into three groups based on to organic C loading, PO${}_{4}^{3-}$, and Fe^2+^ are somewhat consistent with those of Lisa et al. (2017), who reported that porewater Fe^2+^, NO_x_ concentrations and sediment organics (they did not measure PO${}_{4}^{3-}$) were the physico-chemical factors most strongly correlated with the composition of the *nirS* denitrifying microbial communities in a shallow temperate estuary. On a global scale, the analyses from Jones and Hallin (2010) report that the relatedness of *nirS* communities were negatively correlated with NH${}_{4}^{+}$. Our results are also consistent with the findings of Graf et al. (2014) who showed that *nirS* denitrifying communities are strongly shaped by their habitat.

DNRA rates were positively correlated with organic C loading, which has been shown previously to control the partitioning of denitrification and DNRA (Wiegner and Seitzinger, 2004; Rahman et al., 2019). The regression models from Kessler et al. (2018) also revealed that organic C loading was a major predictor for DNRA rates. These results complement the findings from a number of studies which highlight that microbes exhibit substrate preferences for the size and age of OM (Findlay et al., 2003; Ding et al., 2015; Wang et al., 2015). The authors from these studies indicate that the quantity and more importantly, the quality of OM is important in structuring niche partitioning and microbial diversity gradients. The quality of OM and the links between microbial community structure and their associated N fluxes should be investigated more intensively in this and other systems (Bending et al., 2002).

### Functional Communities and Networks

Our second hypothesis suggested that based on the functional genes catalyzing nitrite reduction during denitrification and DNRA pathways we could differentiate between estuaries with a low and high N removal capacity. When we compared the beta-diversity of the denitrifying communities between estuaries with low and high N removal capacity the ANOSIM R values were the lowest (yet still significant) compared to the different land uses and at a per estuary basis comparison. These results indicated that in this data set, the information from functional N-cycling genes at a community level did not provide us with enough information to differentiate between estuaries that signaled high or low N removal. These conclusions remained across the two different similarity thresholds, zOTUs and 88% similarity.

Co-occurrence network analyses (Brodland, 2015) were used to explore the structure of the denitrifying and DNRA communities across a gradient of anthropogenic pressure (Sabater et al., 2007; Qu et al., 2017). We tested the hypothesis that networks analysis would show greater niche partitioning, exhibited as a greater number of modular components in the network, in the pristine sites compared to urban sites (De Menezes et al., 2015; Lurgi et al., 2019). The underlying argument was that human disturbance would create a more uniform environment in the urban sites (resulting in a lower modularity and smaller niche partitioning) compared to the pristine sites. We postulated that a greater number of modular components (greater niche partitioning) in the pristine sites could reveal different life strategies across species. Our network analyses for the *nirS* sequences showed that the modularity for the denitrifying communities in the urban sites was considerably lower compared to the pristine sites. In the urban sites the majority of the denitrifying OTUs were clumped and linked, suggesting a high similarity in the environmental responses between these OTUs (and their lifestyles). These results suggest that most of the denitrifying species in the urban sites are selected by environmental parameters (high anthropogenic pressures such as nutrient loadings). Overall our data show that the anthropogenically impacted sites, based on the *nirS* network topology, resulted in a more homogeneous denitrifying community.

Qu et al. (2017) showed that anthropogenic inputs resulted in a decrease of functional genes in general compared to a more pristine site, suggesting high functional gene redundancy in more urban areas. In our data set, for the functional *nirS* gene we suggest a functional redundancy in the life strategies of the denitrifying organisms in the urban sites. An open question, however, in this dataset and for microbial ecology in general is why there remains such a high alpha diversity within the same function? The pristine sites, with a greater environmental complexity, structured the denitrifying community in a more diverse manner (a higher degree of niche partitioning). The pristine sites will be subjected to natural environmental fluctuations, which could lead to the coexistence of denitrifying species with contrasting nutrient affinities (Martens-Habbena et al., 2009) and denitrifying species with different life styles which can thrive under a range of nutrient concentrations (Bowen et al., 2011). In addition, we suggest that in the pristine sites both environment and biotic interactions are more important for the denitrifying network structure, whereas in the urban sites the continuous input of nutrients shaped the *nirS* denitrifying community to a more uniform structure.

The *nrfA* network topology did not reveal strong differences in the modularities compared to the *nirS* networks. The larger alpha diversity for the *nrfA* gene might be at the base of the similarities between the different modularities. The fact that the *nirS* gene provide us with insights into N removal (Francis et al., 2013; Zheng et al., 2015), along with the distinctive differences in the modularities, the number of keystone OTUs in the microbial networks between the urban and pristine sites suggest that the *nirS* gene could be a likely gene candidate to understand the mechanisms by which these denitrifying communities form and respond to anthropogenic pressures.

### Regression Models

Our third hypothesis suggested that the linear regression models from Kessler et al. (2018) would improve when we integrate bacterial biomass and functional N gene copy data. Kessler et al. (2018) investigated the key controls over the relative rates of N removal via denitrification and DNRA presented in this study. The authors found that when nitrate was limiting, when sediments were highly reducing and when large concentrations of dissolved Fe^2+^ were present, estuaries would be driven toward being N recycling rather than N removing. When we integrated molecular data including *16S*, *nirS*, and *nrfA* abundances and their ratios with the best predictor variables from Kessler et al. (2018), we noted slight improvements in the regression models. However, if we use an *a priori* factor [in this case the lowest BIC factor from the model runs by Kessler et al. (2018)], we did not see any improvements compared to the models from Kessler et al. (2018), Zhang et al. (2018) also used multiple regression models to predict benthic N-loss activities with environmental factors and functional microbial gene-based data and reported that the gene abundances from their study were poorly correlated with N losses. Overall they found that chlorophyll *a* in the bottom waters and sediment organic C content were still the best predictors. Zhang et al. (2018), however, noted that the ratios of different functional genes increased the predictive powers of the regression models for total N loss. Again, in our study, we did not see an overall improvement when we added the ratios of different functional genes. Attard et al. (2011) also noted that denitrification rates in particular and N-cycling rates in general (Graham et al., 2014) were primarily controlled by abiotic soil parameters and that microbial community structure or functional composition did not improve the predictive capacities of their models. Integrating microbial trait data into (ecosystem) models remains a challenge (Treseder et al., 2012; Zhang et al., 2018), yet a number of studies highlight promising results in predicting elemental fluxes when microbial traits, including microbial biomass, were explicitly considered (Graham et al., 2016; Louca et al., 2016; Pommier et al., 2018; Yu and Zhuang, 2019). The latter encourage further research to investigate how the added value of microbial data may increase our ability to predict ecosystem processes. Our data suggest that the microbiomes, which are shaped by their environments in a predictable way, play a key role on the controls of the individual N removal rates in the 11 estuaries. It is clear that further research is required to explore how the bacterial community composition can be used in providing qualitative and quantitative information on the fate of nitrogen in estuaries.

## Data Availability Statement

Sequence data have been submitted to the National Center for Biotechnology Information under bioproject ID: PRJNA61138.

## Author Contributions

ER and BH executed qPCRs. ER, KK, AB, and LB analyzed the data and wrote the manuscript. AK and PC developed the experimental design and measured the rates. JK did the library preps and contributed to the data analyses and interpretation. AB analyzed the sequence data. All authors helped contributed to the data analysis, interpretation, and with the editing of the manuscript.

## Conflict of Interest

The authors declare that the research was conducted in the absence of any commercial or financial relationships that could be construed as a potential conflict of interest.

## References

[B1] Attard E. Recous S. Chabbi A. De Berranger C. Guillaumaud N. Labreuche J. (2011). Soil environmental conditions rather than denitrifier abundance and diversity drive potential denitrification after changes in land uses. *Glob. Change Biol.* 17 1975–1989. 10.1111/j.1365-2486.2010.02340.x

[B2] Bending G. D. Turner M. K. Jones J. E. (2002). Interactions between crop residue and soil organic matter quality and the functional diversity of soil microbial communities. *Soil Biol. Biochem.* 34 1073–1082. 10.1016/s0038-0717(02)00040-8

[B3] Bowen J. Byrnes J. Weisman D. Colaneri C. (2013). Functional gene pyrosequencing and network analysis: an approach to examine the response of denitrifying bacteria to increased nitrogen supply in salt marsh sediments. *Front. Microbiol.* 4:342. 10.3389/fmicb.2013.00342 24348464 PMC3841915

[B4] Bowen J. L. Ward B. B. Morrison H. G. Hobbie J. E. Valiela I. Deegan L. A. (2011). Microbial community composition in sediments resists perturbation by nutrient enrichment. *ISME J.* 5:1540. 10.1038/ismej.2011.22 21412346 PMC3160680

[B5] Brodland G. W. (2015). How computational models can help unlock biological systems. *Sem. Cell Dev. Biol.* 47 62–73. 10.1016/j.semcdb.2015.07.001 26165820

[B6] Brown M. B. (1975). A method for combining non-independent, one-sided tests of significance. *Biometrics* 31 987–992.

[B7] Brunet R. Garcia-Gil L. (1996). Sulfide-induced dissimilatory nitrate reduction to ammonia in anaerobic freshwater sediments. *FEMS Microbiol. Ecol.* 21 131–138. 10.1111/j.1574-6941.1996.tb00340.x

[B8] Buchfink B. Xie C. Huson D. H. (2014). Fast and sensitive protein alignment using DIAMOND. *Nat. Methods* 12:59. 10.1038/nmeth.3176 25402007

[B9] Burgin A. J. Hamilton S. K. (2007). Have we overemphasized the role of denitrification in aquatic ecosystems? A review of nitrate removal pathways. *Front. Ecol. Environ.* 5:89–96. 10.1890/1540-9295(2007)5[89:hwotro]2.0.co;2

[B10] Callahan B. J. McMurdie P. J. Holmes S. P. (2017). Exact sequence variants should replace operational taxonomic units in marker-gene data analysis. *ISME J.* 11:2639. 10.1038/ismej.2017.119 28731476 PMC5702726

[B11] Caro-Quintero A. Ochman H. (2015). Assessing the unseen bacterial diversity in microbial communities. *Genome Biol. Evol.* 7 3416–3425. 10.1093/gbe/evv234 26615218 PMC4700968

[B12] Crawley M. J. (2012). *The R Book.* Hoboken, NJ: John Wiley & Sons.

[B13] Csardi G. Nepusz T. (2006). The igraph software package for complex network research. *Inter J. Comp. Syst.* 1695 1–9.

[B14] Dalsgaard T. Bak F. (1994). Nitrate reduction in a sulfate-reducing bacterium, Desulfovibrio desulfuricans, isolated from rice paddy soil: sulfide inhibition, kinetics, and regulation. *Appl. Environ. Microbiol.* 60 291–297. 10.1128/aem.60.1.291-297.1994 16349159 PMC201302

[B15] Damashek J. Francis C. A. (2018). Microbial nitrogen cycling in estuaries: from genes to ecosystem processes. *Estua. Coas.* 41 626–660. 10.1007/s12237-017-0306-2

[B16] De Menezes A. B. Prendergast−Miller M. T. Richardson A. E. Toscas P. Farrell M. Macdonald L. M. (2015). Network analysis reveals that bacteria and fungi form modules that correlate independently with soil parameters. *Environ. Microbiol.* 17 2677–2689. 10.1111/1462-2920.12559 25040229

[B17] Diaz R. J. Rosenberg R. (2008). Spreading dead zones and consequences for marine ecosystems. *Science* 321 926–929. 10.1126/science.1156401 18703733

[B18] Ding J. Zhang Y. Wang M. Sun X. Cong J. Li D. (2015). Soil organic matter quantity and quality shape microbial community compositions of subtropical broadleaved forests. *Mol. Ecol.* 24 5175–5185. 10.1111/mec.13384 26363284

[B19] Edgar R. C. (2010). Search and clustering orders of magnitude faster than BLAST. *Bioinformatics* 26 2460–2461. 10.1093/bioinformatics/btq461 20709691

[B20] Edgar R. C. Flyvbjerg H. (2015). Error filtering, pair assembly and error correction for next-generation sequencing reads. *Bioinformatics* 31 3476–3482. 10.1093/bioinformatics/btv401 26139637

[B21] Epskamp S. Cramer A. O. Waldorp L. J. Schmittmann V. D. Borsboom D. (2012). qgraph: Network visualizations of relationships in psychometric data. *J. Statist. Softw.* 48 1–18.

[B22] Faust K. Raes J. (2016). CoNet app: inference of biological association networks using Cytoscape. *F1000Res.* 5 1519–1519. 10.12688/f1000research.9050.2 27853510 PMC5089131

[B23] Faust K. Sathirapongsasuti J. F. Izard J. Segata N. Gevers D. Raes J. (2012). Microbial co-occurrence relationships in the human microbiome. *PLoS Comput. Biol.* 8:e1002606. 10.1371/journal.pcbi.1002606 22807668 PMC3395616

[B24] Findlay S. E. Sinsabaugh R. L. Sobczak W. V. Hoostal M. (2003). Metabolic and structural response of hyporheic microbial communities to variations in supply of dissolved organic matter. *Limnol. Oceanogr.* 48 1608–1617. 10.4319/lo.2003.48.4.1608

[B25] Francis C. O’Mullan G. Cornwell J. Ward B. (2013). Transitions in nirS-type denitrifier diversity, community composition, and biogeochemical activity along the Chesapeake Bay estuary. *Front. Microbiol.* 4:237. 10.3389/fmicb.2013.00237 24009603 PMC3757304

[B26] Giblin A. E. Tobias C. R. Song B. Weston N. Banta G. T. Rivera-Monroy V. (2013). The importance of dissimilatory nitrate reduction to ammonium (DNRA) in the nitrogen cycle of coastal ecosystems. *Oceanography* 26 124–131. 10.5670/oceanog.2013.54

[B27] Graf D. R. Jones C. M. Hallin S. (2014). Intergenomic comparisons highlight modularity of the denitrification pathway and underpin the importance of community structure for N2O emissions. *PLoS One* 9:e114118. 10.1371/journal.pone.0114118 25436772 PMC4250227

[B28] Graham E. B. Knelman J. E. Schindlbacher A. Siciliano S. Breulmann M. Yannarell A. (2016). Microbes as engines of ecosystem function: when does community structure enhance predictions of ecosystem processes? *Front. Microbiol.* 7:214. 10.3389/fmicb.2016.00214 PMC4764795 26941732

[B29] Graham E. B. Wieder W. R. Leff J. W. Weintraub S. R. Townsend A. R. Cleveland C. C. (2014). Do we need to understand microbial communities to predict ecosystem function? A comparison of statistical models of nitrogen cycling processes. *Soil Biol. Biochem.* 68 279–282. 10.1016/j.soilbio.2013.08.023

[B30] Greaver T. Clark C. Compton J. Vallano D. Talhelm A. Weaver C. (2016). Key ecological responses to nitrogen are altered by climate change. *Nat. Clim. Change* 6:836 10.1038/nclimate3088

[B31] Holm S. (1979). A simple sequentially rejective multiple test procedure. *Scand. J. Statist.* 6 65–70.

[B32] Howarth R. Swaney D. Billen G. Garnier J. Hong B. Marino R. (2012). Nitrogen fluxes from the landscape are controlled by net anthropogenic nitrogen inputs and by climate. *Front. Ecol. Environ.* 10:37–43. 10.1890/100178

[B33] Huson D. H. Auch A. F. Qi J. Schuster S. C. (2007). MEGAN analysis of metagenomic data. *Genome Res.* 17 377–386. 10.1101/gr.5969107 17255551 PMC1800929

[B34] Jones C. M. Hallin S. (2010). Ecological and evolutionary factors underlying global and local assembly of denitrifier communities. *ISME J.* 4:633. 10.1038/ismej.2009.152 20090785

[B35] Kessler A. J. Roberts K. L. Bissett A. Cook P. L. (2018). Biogeochemical controls on the relative importance of denitrification and dissimilatory nitrate reduction to ammonium in estuaries. *Glob. Biogeochem. Cycles* 32 1045–1057. 10.1029/2018gb005908

[B36] Lee J. A. Francis C. A. (2017). Spatiotemporal characterization of san francisco bay denitrifying communities: a comparison of nirK and nirS diversity and abundance. *Microb. Ecol.* 73 271–284. 10.1007/s00248-016-0865-y 27709247

[B37] Lisa J. A. Jayakumar A. Ward B. B. Song B. (2017). nirS-type denitrifying bacterial assemblages respond to environmental conditions of a shallow estuary. *Environ. Microbiol. Rep.* 9 766–778. 10.1111/1758-2229.12594 28914491

[B38] Louca S. Hawley A. K. Katsev S. Torres-Beltran M. Bhatia M. P. Kheirandish S. (2016). Integrating biogeochemistry with multiomic sequence information in a model oxygen minimum zone. *Proc. Natl. Acad. Sci. U.S.A.* 113 E5925–E5933. 27655888 10.1073/pnas.1602897113 PMC5056048

[B39] Lurgi M. Thomas T. Wemheuer B. Webster N. S. Montoya J. M. (2019). Modularity and predicted functions of the global sponge-microbiome network. *Nat. Commun.* 10:992. 10.1038/s41467-019-08925-4 PMC6397258 30824706

[B40] Magoč T. Salzberg S. L. (2011). FLASH: fast length adjustment of short reads to improve genome assemblies. *Bioinformatics* 27 2957–2963. 10.1093/bioinformatics/btr507 21903629 PMC3198573

[B41] Martens-Habbena W. Berube P. M. Urakawa H. José R. Stahl D. A. (2009). Ammonia oxidation kinetics determine niche separation of nitrifying archaea and bacteria. *Nature* 461:976. 10.1038/nature08465 19794413

[B42] Michotey V. Méjean V. Bonin P. (2000). Comparison of methods for quantification of cytochrome cd 1-denitrifying bacteria in environmental marine samples. *Appl. Environ. Microbiol.* 66 1564–1571. 10.1128/aem.66.4.1564-1571.2000 10742243 PMC92024

[B43] Mohan S. B. Schmid M. Jetten M. Cole J. (2004). Detection and widespread distribution of the nrfA gene encoding nitrite reduction to ammonia, a short circuit in the biological nitrogen cycle that competes with denitrification. *FEMS Microbiol. Ecol.* 49 433–443. 10.1016/j.femsec.2004.04.012 19712292

[B44] Muyzer G. De Waal E. C. Uitterlinden A. G. (1993). Profiling of complex microbial populations by denaturing gradient gel electrophoresis analysis of polymerase chain reaction-amplified genes coding for 16S rRNA. *Appl. Environ. Microbiol.* 59 695–700. 10.1128/aem.59.3.695-700.1993 7683183 PMC202176

[B45] Oksanen J. Kindt R. Legendre P. O’Hara B. Stevens M. H. H. Oksanen M. J. (2007). The vegan package. *Commun. Ecol. Pack.* 10 631–637.

[B46] Piehler M. Smyth A. (2011). Habitat−specific distinctions in estuarine denitrification affect both ecosystem function and services. *Ecosphere* 2 1–17.

[B47] Pommier T. Cantarel A. A. Grigulis K. Lavorel S. Legay N. Baxendale C. (2018). The added value of including key microbial traits to determine nitrogen−related ecosystem services in managed grasslands. *J. Appl. Ecol.* 55 49–58. 10.1111/1365-2664.13010

[B48] Qu X. Ren Z. Zhang H. Zhang M. Zhang Y. Liu X. (2017). Influences of anthropogenic land use on microbial community structure and functional potentials of stream benthic biofilms. *Sci. Rep.* 7:15117. 10.1038/s41598-017-15624-x PMC5678132 29118402

[B49] R Core Team (2013). *R: A Language and Environment for Statistical Computing.* Vienna: R Core Team.

[B50] Rahman M. M. Roberts K. L. Grace M. R. Kessler A. J. Cook P. L. (2019). Role of organic carbon, nitrate and ferrous iron on the partitioning between denitrification and DNRA in constructed stormwater urban wetlands. *Sci. Total Environ.* 666 608–617. 10.1016/j.scitotenv.2019.02.225 30807951

[B51] Roberts K. L. Kessler A. J. Grace M. R. Cook P. L. (2014). Increased rates of dissimilatory nitrate reduction to ammonium (DNRA) under oxic conditions in a periodically hypoxic estuary. *Geochim. Cosmochim. Acta* 133 313–324. 10.1016/j.gca.2014.02.042

[B52] Robertson E. K. Roberts K. L. Burdorf L. D. Cook P. Thamdrup B. (2016). Dissimilatory nitrate reduction to ammonium coupled to Fe (II) oxidation in sediments of a periodically hypoxic estuary. *Limnol. Oceanogr.* 61 365–381. 10.1002/lno.10220

[B53] Sabater S. Guasch H. Ricart M. Romaní A. Vidal G. Klünder C. (2007). Monitoring the effect of chemicals on biological communities. The biofilm as an interface. *Anal. Bioanal. Chem.* 387 1425–1434. 10.1007/s00216-006-1051-8 17225111

[B54] Seitzinger S. Mayorga E. Bouwman A. Kroeze C. Beusen A. Billen G. (2010). Global river nutrient export: a scenario analysis of past and future trends. *Glob. Biogeochem. Cycles* 24.

[B55] Small C. Cohen J. (2004). Continental physiography, climate, and the global distribution of human population. *Curr. Anthropol.* 45 269–277. 10.1086/382255

[B56] Tapolczai K. Vasselon V. Bouchez A. Stenger−Kovács C. Padisák J. Rimet F. (2019). The impact of OTU sequence similarity threshold on diatom−based bioassessment: a case study of the rivers of Mayotte (France, Indian Ocean). *Ecol. Evol.* 9 166–179. 10.1002/ece3.4701 30680104 PMC6342121

[B57] Throbäck I. N. Enwall K. Jarvis Å Hallin S. (2004). Reassessing PCR primers targeting nirS, nirK and nosZ genes for community surveys of denitrifying bacteria with DGGE. *FEMS Microbiol. Ecol.* 49 401–417. 10.1016/j.femsec.2004.04.011 19712290

[B58] Tiedje J. M. Sexstone A. J. Myrold D. D. Robinson J. A. (1983). Denitrification: ecological niches, competition and survival. *Antonie Van Leeuwenhoek* 48 569–583. 10.1007/bf00399542 6762848

[B59] Treseder K. K. Balser T. C. Bradford M. A. Brodie E. L. Dubinsky E. A. Eviner V. T. (2012). Integrating microbial ecology into ecosystem models: challenges and priorities. *Biogeochemistry* 109 7–18. 10.1007/s10533-011-9636-5

[B60] Wang H. Boutton T. W. Xu W. Hu G. Jiang P. Bai E. (2015). Quality of fresh organic matter affects priming of soil organic matter and substrate utilization patterns of microbes. *Sci. Rep.* 5:10102. 10.1038/srep10102 PMC4426597 25960162

[B61] Welsh A. Chee-Sanford J. Connor L. Löffler F. Sanford R. (2014). Refined NrfA phylogeny improves PCR-based nrfA gene detection. *Appl. Environ. Microbiol.* 80 2110–2119. 10.1128/aem.03443-13 24463965 PMC3993153

[B62] Wiegner T. N. Seitzinger S. P. (2004). Seasonal bioavailability of dissolved organic carbon and nitrogen from pristine and polluted freshwater wetlands. *Limnol. Oceanogr.* 49 1703–1712. 10.4319/lo.2004.49.5.1703

[B63] Yu C. Huang X. Chen H. Godfray H. C. J. Wright J. S. Hall J. W. (2019). Managing nitrogen to restore water quality in China. *Nature* 567 516–520. 10.1038/s41586-019-1001-1 30818324

[B64] Yu T. Zhuang Q. (2019). Quantifying global N 2 O emissions from natural ecosystem soils using trait-based biogeochemistry models. *Biogeosciences* 16 207–222. 10.5194/bg-16-207-2019

[B65] Zhang X. Zhang Q. Yang A. Hou L. Zheng Y. Zhai W. (2018). Incorporation of microbial functional traits in biogeochemistry models provides better estimations of benthic denitrification and anammox rates in coastal oceans. *J. Geophys. Res. Biogeosciences* 123 3331–3352. 10.1029/2018jg004682

[B66] Zheng Y. Hou L. Liu M. Gao J. Yin G. Li X. (2015). Diversity, abundance, and distribution of nirS-harboring denitrifiers in intertidal sediments of the Yangtze Estuary. *Microb. Ecol.* 70 30–40. 10.1007/s00248-015-0567-x 25592637

