# Supplementary material for: Can We Use Functional Genetics to Predict the Fate of Nitrogen in Estuaries?
Source: Front Microbiol. 2020 Jun 11;11:1261. doi: 10.3389/fmicb.2020.01261 (PMC7325967; doi:10.3389/fmicb.2020.01261)
Supplement: Supplementary file 1 [file Data_Sheet_1.DOCX]

Supplementary Material

Can we use functional genetics to predict the fate of nitrogen in estuaries?

Eric J. Raes^1*^, Kristen Karsh^1^, Adam J. Kessler^2^, Perran L.M. Cook^3^, Bronwyn Holmes^1^, Jodie van de Kamp^1^, Lev Bodrossy^1^, Andrew Bissett^1^

1. CSIRO Oceans and Atmosphere, Hobart, Australia, 7000
2. School of Earth, Atmosphere and Environment, Monash University, Australia, 3800
3. Water Studies Centre, School of Chemistry, Monash University, Australia, 3800

*** Correspondence:**Eric J. Raes
eric.raes@csiro.au

Keywords: Denitrification; DNRA; Functional genes; Estuary; Co-occurrence; Nitrogen

## Supplementary Tables and Figures

Water quality, sampling methodology and physical parameters along with denitrification and dissimilatory nitrate reduction to ammonium (DNRA) rates were measured in eleven estuaries in Victoria, Australia. Detailed information can be found in the manuscript and supplementary material from Kessler et al. (2018) <https://agupubs.onlinelibrary.wiley.com/doi/full/10.1029/2018GB005908>.

Rarefaction curves for the *nirS* and *nrfA* amplicon sequence data.

**
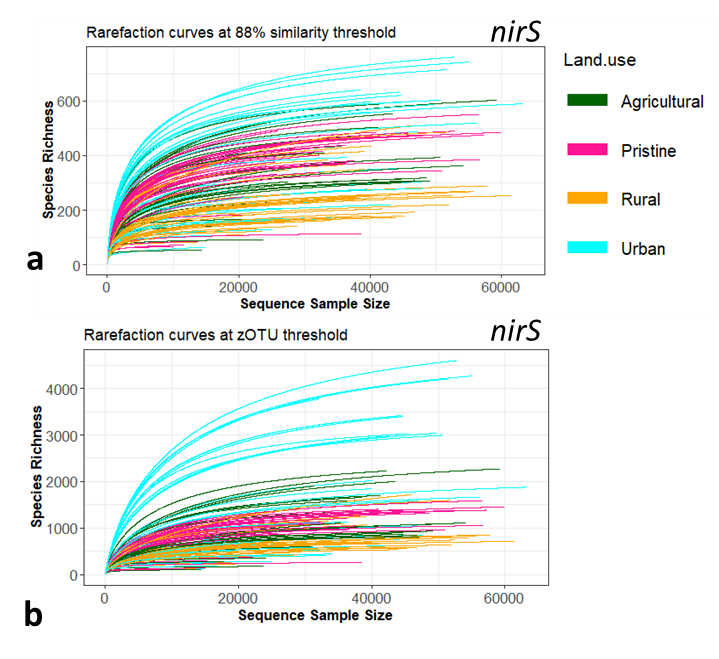
**

**Supplementary Figure 1:** Rarefaction curves for the *nirS* sequence data; **a)** for the sequences clustered at the 88% similarity threshold and **b)** for the sequences clustered at a zOTU threshold. Samples are colour coded according to their land use.

**
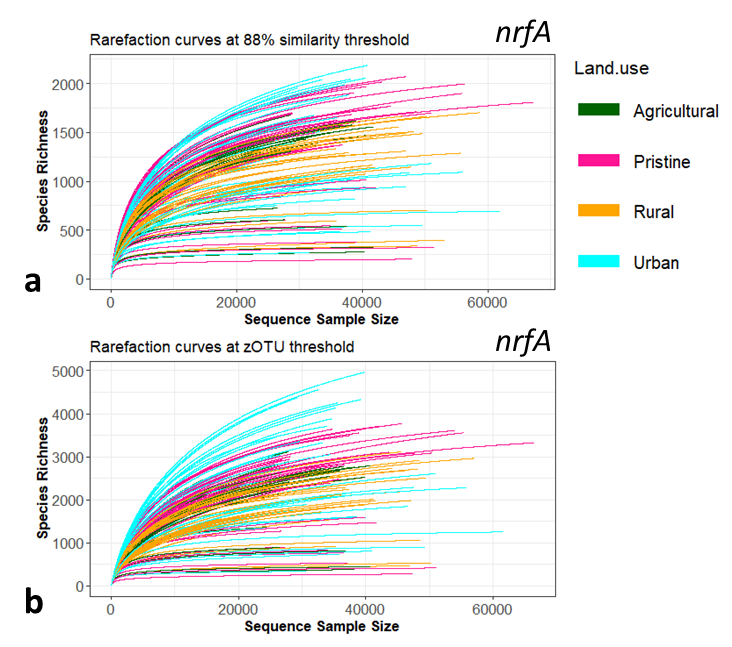
**

**Supplementary Figure 2:** Rarefaction curves for the *nrfA* sequence data; **a)** for the sequences clustered at the 88% similarity threshold and **b)** for the sequences clustered at a zOTU threshold. Samples are colour coded according to their land use.

**Supplementary** **Table 1** is attached as a separate Excel file.

**Supplementary Table 2:** The 24 physio-chemical parameters used to make PCoA figure.

Latitude (Lats), longitude (Longs), percentage of catchment area fertilized (fert.pc), population per km^2^ of catchment area (pop.dens), estuary area (Area in m^2^), nitrate + nitrite (NOx load in mg/km^2^/yr), total nitrogen (TN load in mg/km^2^/yr), total phosphorus (TP in mg/km^2^/yr), total suspended solids (TSS in mg/km^2^/yr), iron (Fe^2+^ in µmol/m^2^), ascorbate extractible Fe^2+^ (Fe.asc in µmol/m^2^), PO_4_^3-^(P in µmol/m^2^), ascorbate extractible (bound) phosphorus (P.asc in µmol/m^2^), acid volatile sulphide ~= FeS (AVS in µmol/m^2^), ammonium (A in µmol/m^2^), organic carbon (C in µmol/m^2^), Temperature (T in ^o^C), , dissolved oxygen saturation (DO, % air saturation), salinity (Sal in PSU), overlying water NOx concentration ([NOx]_OW_ in μmol/L), overlying water NH_4_^+^ concentration ([NH_4_^+^]_OW_ in μmol/L), overlying water filterable reactive phosphorus (FRP in in μmol/L), pore water FRP (in mmol/m^2^ integrated over depth), pore water Fe^2+^ (in mmol/m^2^ integrated over depth) and pore water ascorbate extractible Fe^2+^ (Fe.asc in mmol/m^2^ integrated over depth).


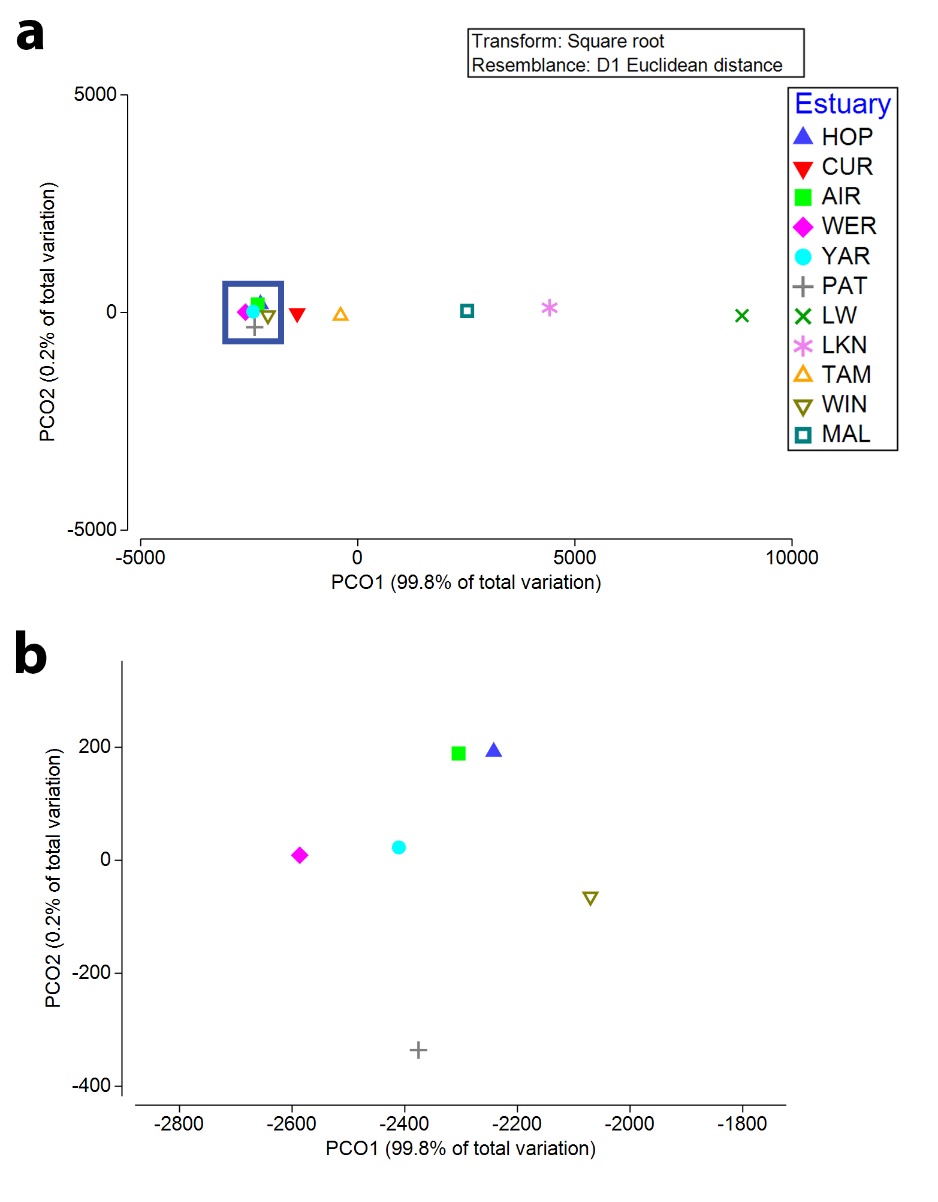


**Supplementary Figure 3:** Principal coordinate analysis (PCoA) with Bray-Curtis dissimilarity of 24 parameters for 11 estuaries along the Victorian coastline. The 24 physio-chemical parameters (see supplementary table 1) were square root transformed. **b)** is a zoom from the blue box on **a).**

**Supplementary Table 3:**  Average dissimilarity percentages for two N-cycling amplicons among 11 estuaries at a zOTU and at 88% similarity level derived from Similarity Percentage analyses (SIMPER).

|  | **Dissimilarity (%)** | |
| --- | --- | --- |
| **Amplicon** | **zOTU** | **88%** |
| ***nirS*** | 89.2 ±7.7 | 77.8 ±9.5 |
| ***nrfA*** | 92.7 ±4.4 | 82.0 ±6.4 |

Average ±SD for all pairwise comparisons between 11 estuaries.

**
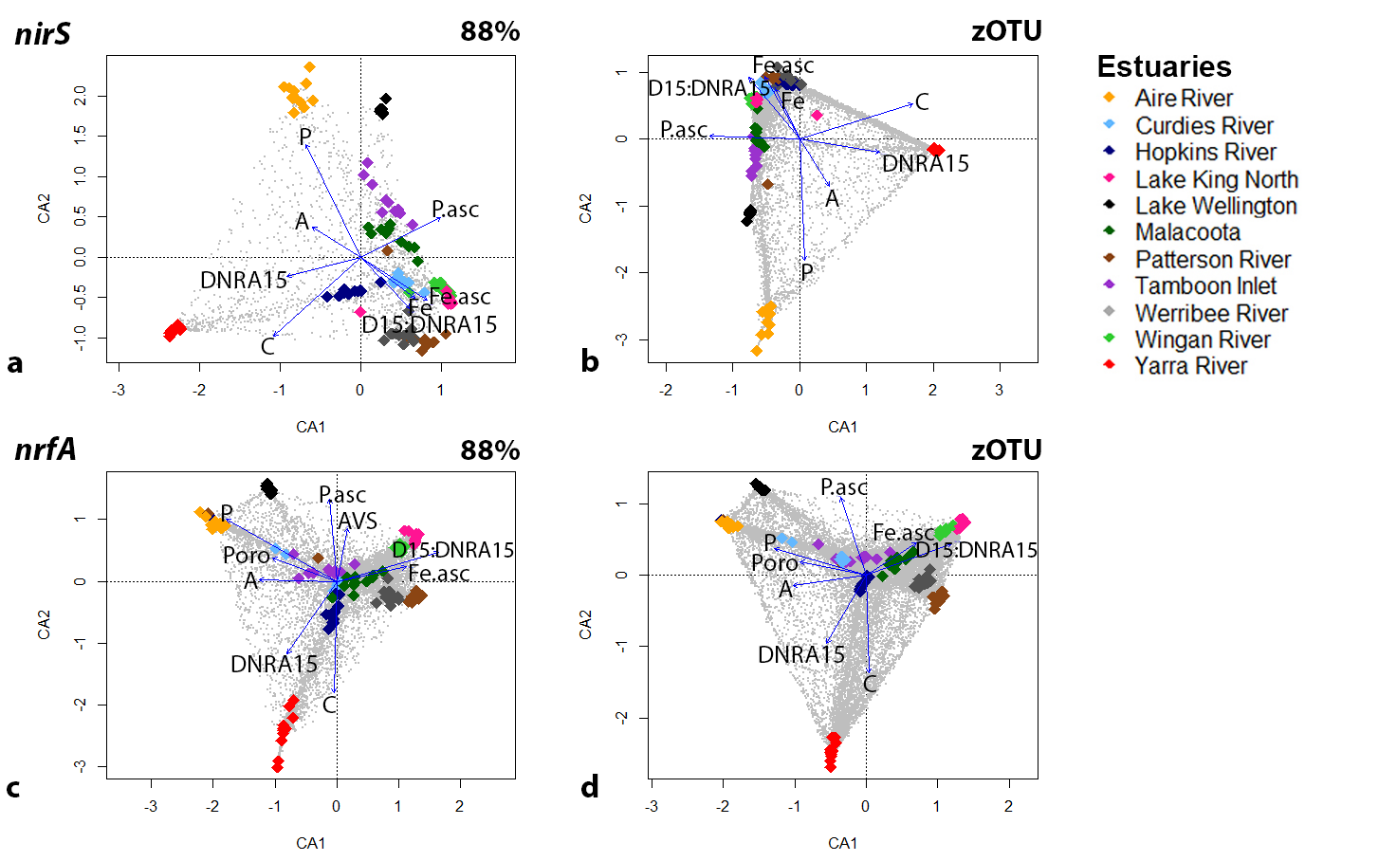
**

**Supplementary Figure 4:** Canonical correspondence analysis (CCA) for the *nirS* gene using OTUs at the 88% similarity **(a)** and zOTU threshold **(b)** and for the *nrfA* gene using OTUs at the 88% similarity **(c)** and zOTU threshold **(c)**. Amplicon data were Hellinger transformed to decrease the contribution of abundant species and environmental parameters were standardized with the ‘standardize’ function using decostand from the Vegan Package (Oksanen et al., 2007). Significant (p<0.05) environmental parameters were derived using the envfit function in Vegan and overlaid as vectors. Environmental parameters from all six depths were: sediment ascorbate-extractible Fe^2+^ concentrations (Fe.asc in µM); Ascorbate extractible (bound) PO_4_^3-^ (P.asc in µM); Porosity (Poro); NH_4_^+^ in pore water (A in µM); Organic carbon (C in µM); Filterable reactive phosphorus in pore water (P in µM), Acid volatile sulphide (AVS in µM). Colours represent estuaries and grey dots are the OTUs.

**
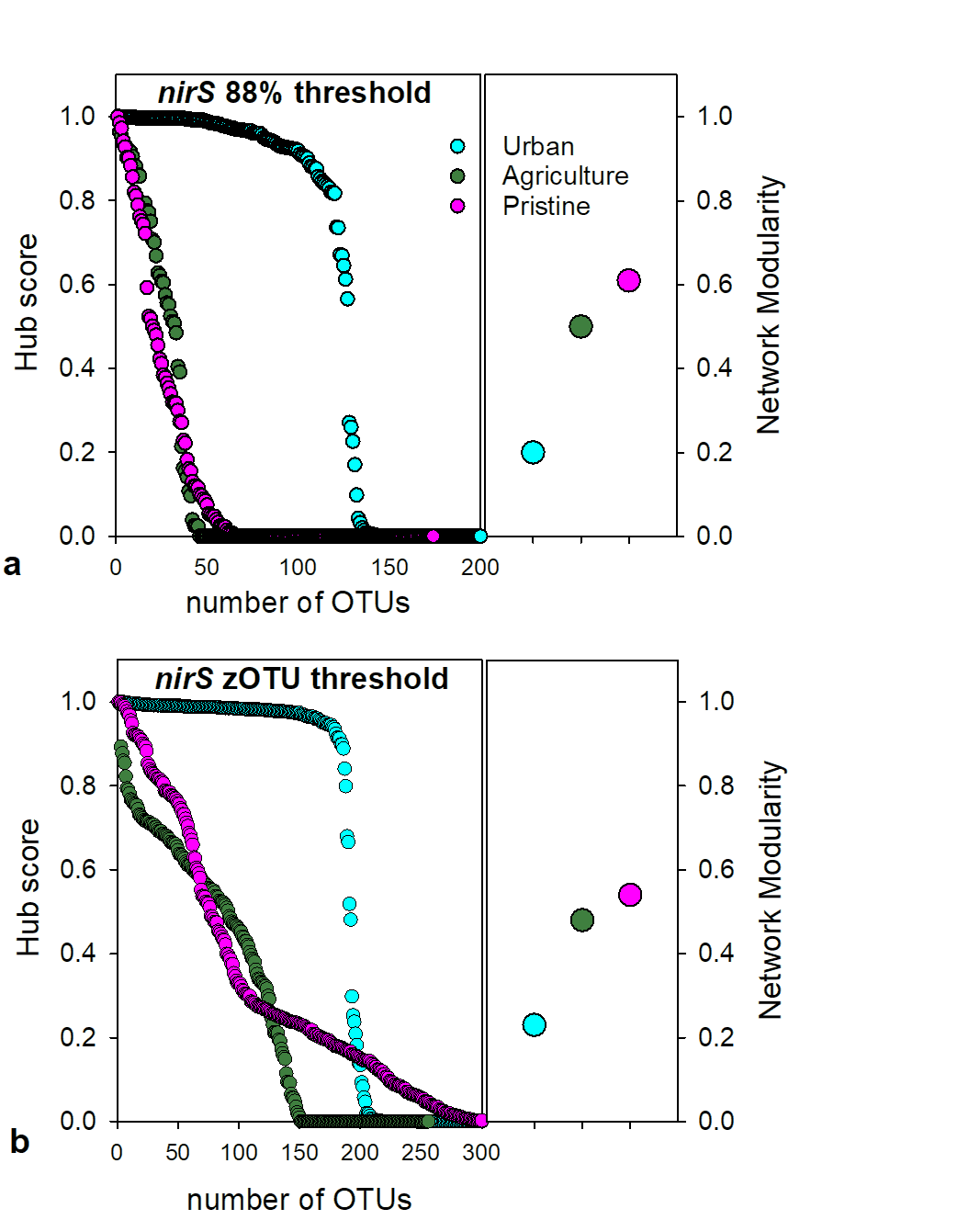
**

**Supplementary Figure 5:** Hubs scores for derived from microbiome networks based on *nirS* OTUs clustered at the 88% similarity threshold (a) and the zOTU threshold (b) across Urban, Agricultural and Pristine land uses. Hub scores are displayed on the left panels and network modularities on the right panels.


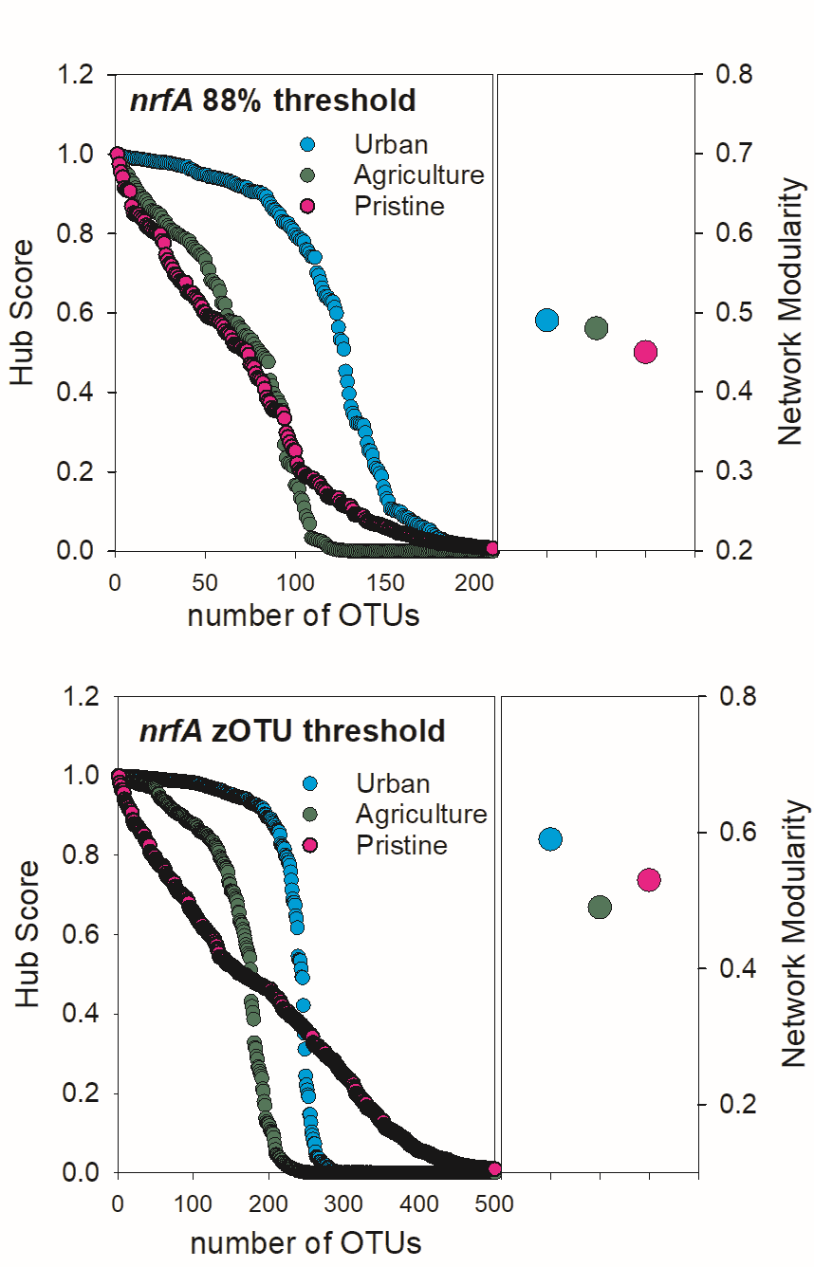


**Supplementary Figure 6:** Hubs scores for derived from microbiome networks based on *nrfA* OTUs clustered at the 88% similarity threshold (a) and the zOTU threshold (b) across Urban, Agricultural and Pristine land uses. Hub scores are displayed on the left panels and network modularities on the right panels.

**Table 4:** Best models based on BIC for potential denitrification rates (D_15_), DNRA rates (DNRA_15_) and the D_15_/DNRA_15_ ratio in individual slices. Grey shaded block are the models from Kessler et al. (2018); White block are the models including *16S* DNA, *nirS* and *nrfA* qPCR data from this study. ‘No model improvement’ means that the addition of the functional gene predictor data only showed up no more than twice in the top 20 models.

|  | **y** | **predictor 1** | **predictor 2** | **predictor 3** | **BIC** | **AIC_c_** | **p** | **R^2^** | **n** |
| --- | --- | --- | --- | --- | --- | --- | --- | --- | --- |
| **ln (D_15_) =** | -1.55 | -1.245 ln (d_sed_) | +9.99 x 10^-6^ [AVS] |  | 124 | 118 | <0.001 | 0.757 | 46 |
| **ln (D_15_) + *16S*** |  | No | Model | Improvement |  |  |  |  | 45 |
| **ln (D_15_) + *nirS*** |  | No | Model | Improvement |  |  |  |  | 45 |
| **ln (D_15_) + *nrfA*** |  | No | Model | Improvement |  |  |  |  | 33 |
| **ln (D_15_) + ln(*nirS*:*nrfA*)** |  | No | Model | Improvement |  |  |  |  | 33 |
| **ln (D_15_) + ln(*nirS*:*16S*)** |  | No | Model | Improvement |  |  |  |  | 45 |
| **ln (D_15_) + ln(*nrfA:16S*)** |  | No | Model | Improvement |  |  |  |  | 33 |
| **ln (DNRA_15_) =** | -2.60 | -0.931 ln (d_sed_) | +41.8 [C] |  | 110 | 105 | <0.001 | 0.659 | 41 |
| **ln (DNRA_15_) + *16S*** | -9.28 | -1.053 ln (d_sed_) | +44.8 [C] | 0.31 ln *16S* | 111 | 105 | <0.001 | 0.689 | 41 |
| **ln (DNRA_15_) + *nirS*** |  | No | Model | Improvement |  |  |  |  | 39 |
| **ln (DNRA_15_) + *nrfA*** |  | No | Model | Improvement |  |  |  |  | 29 |
| **ln (D_15_) + ln(*nirS*:*nrfA*)** |  | No | Model | Improvement |  |  |  |  | 28 |
| **ln (D_15_) + ln(*nirS*:*16S*)** |  | No | Model | Improvement |  |  |  |  | 39 |
| **ln (D_15_) + ln(*nrfA:16S*)** |  | No | Model | Improvement |  |  |  |  | 29 |
| **ln (D_15_/DNRA_15_) =** | 0.19 | -9.58 x 10^-4^ [NH_4_^+^] | +0.0444 [No_x_]_ow_ | -0.00420 [Fe^2+^] | 84.4 | 79 | <0.001 | 0.508 | 35 |
| **ln (D_15_/DNRA_15_) + *16S*** | 8.19 | -6.701 x 10^-4^ [NH_4_^+^] | +0.0298 [No_x_]_ow_ | -0.38 ln *16S* | 85 | 79 | <0.001 | 0.503 | 35 |
| **ln (D_15_/DNRA_15_) + *nirS*** |  | No | Model | Improvement |  |  |  |  | 34 |
| **ln (D_15_/DNRA_15_) + *nrfA*** |  | No | Model | Improvement |  |  |  |  | 24 |
| **ln (D_15_) + ln(*nirS*:*nrfA*)** |  | No | Model | Improvement |  |  |  |  | 34 |
| **ln (D_15_) + ln(*nirS*:*16S*)** |  | No | Model | Improvement |  |  |  |  | 24 |
| **ln (D_15_) + ln(*nrfA:16S*)** |  | No | Model | Improvement |  |  |  |  | 24 |

LN (D15) = log of denitrification rates; LN (DNRA15), Log of DNRA rates; LN (D15/DNRA15) = log of denitrification to DNRA ratio. Y is the intercept. N = data points.

**
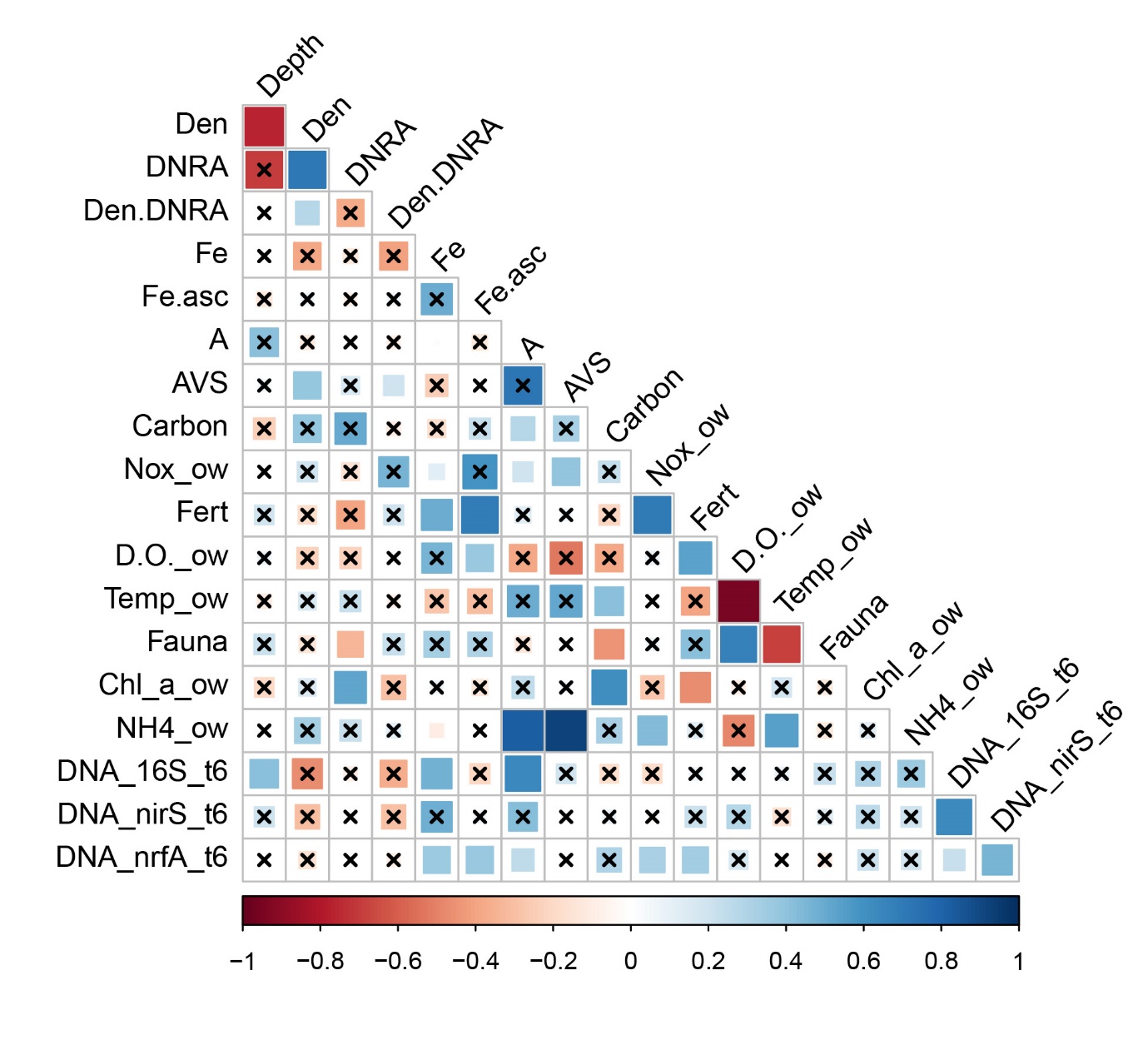
**

**Supplementary Figure 7:** Spearman correlation coefficients (rs) plot for the *16S* rRNA gene and functional N gene abundance, denitrification, DNRA rates and physico-chemical parameters. Den (Denitrification rates), DNRA (dissimilatory nitrate reduction to ammonium rates), Den.DNRA (ratio of Den to DNRA), Depth (in cm), Fe (pore water Fe^2+^ in mmol/m^2^ integrated over depth), Fe.asc (pore water ascorbate extractible Fe^2+^ in mmol/m^2^ integrated over depth), A (NH_4_^+^ in µmol/m^2^), AVS (acid volatile sulphide ~= FeS in µmol/m^2^), Carbon (organic carbon in µmol/m^2^), Nox_ow (overlying water NOx concentration in μmol/L), Fert (percentage of catchment area fertilized), D.O_ow (dissolved oxygen saturation (in overlying water in % air saturation), Temp_ow (Temperature in overlying water in ^o^C), Fauna (Faunal count), Chl_a_ow (chlorophyll-*a* in overlying waters in µg/L), NH4_ow (NH_4_^+^ concentrations in overlying water in μmol/L), DNA_16S_t6, DNA_nirS_t6 and DNA_nrfA_t6 (gene copies per gram wet sediment at time point 6). Positive correlations are displayed in blue and negative correlations in red. Colour intensity and the size of the squares are proportional to the correlation coefficients. P values > 0.05 are indicated by black crosses.

**References:**

Kessler, A. J., Roberts, K. L., Bissett, A., & Cook, P. L. (2018). Biogeochemical controls on the relative importance of denitrification and dissimilatory nitrate reduction to ammonium in estuaries. *Global Biogeochemical Cycles, 32*(7), 1045-1057.

Oksanen, J., Kindt, R., Legendre, P., O’Hara, B., Stevens, M. H. H., Oksanen, M. J., & Suggests, M. (2007). The vegan package. *Community ecology package, 10*, 631-637.
